# Sequencing of aromatase inhibitors

**DOI:** 10.1038/sj.bjc.6602689

**Published:** 2005-08-15

**Authors:** G Bertelli

**Affiliations:** 1 South West Wales Cancer Institute, Sketty, Swansea SA2 8QA, UK

**Keywords:** aromatase inhibitors, sequencing, tamoxifen, cross-resistance

## Abstract

Since the development of the third-generation aromatase inhibitors (AIs), anastrozole, letrozole and exemestane, these agents have been the subject of intensive research to determine their optimal use in advanced breast cancer. Not only have they replaced progestins in second-line therapy and challenged the role of tamoxifen in first-line, but there is also evidence for a lack of cross-resistance between the steroidal and nonsteroidal AIs, meaning that they may be used in sequence to obtain prolonged clinical benefit. Many questions remain, however, as to the best sequence of the two types of AIs and of the other available agents, including tamoxifen and fulvestrant, in different patient groups.

What is the optimal sequence for hormonal therapy in breast cancer? Until recently, the antioestrogen tamoxifen was the only agent approved for adjuvant therapy. At the time of relapse, postmenopausal patients with tamoxifen-resistant disease (progressing during adjuvant tamoxifen or within 1 year from its discontinuation) were generally offered an aromatase inhibitor (AI), followed by a progestin (usually megestrol acetate) when they further progressed. This paradigm needs to be reconsidered in the light of recent developments following the early results of the Arimidex, Tamoxifen Alone or in Combination (ATAC) study (The ATAC Trialists Group, 2002), with anastrozole becoming an alternative to tamoxifen in the early adjuvant setting (years 0–5). Moreover, early results of the MA17 trial (Goss *et al*, 2003a) and of the Intergroup Exemestane Study (Coombes *et al*, 2004) suggest that a sequence of tamoxifen followed by an AI may be beneficial in the early or in the extended adjuvant setting (years 5–10), respectively.

Further results of ongoing adjuvant trials are eagerly awaited, but it is likely that in the future an increasing number of patients who present with advanced breast cancer will already have received an AI (with or without tamoxifen) as part of their adjuvant therapy. The remaining patients will have had tamoxifen only, and a few will be hormone-therapy naïve. After taking into account previous treatments, the choice of an appropriate hormonal therapy should be based on efficacy and tolerability data of the available agents.

Before trying to determine where each agent should fit in the sequence of therapy, it is worth noting that the alternative strategy, combination therapy, does not appear to provide advantages over the use of single agents. The recently completed ATAC study found that the combination of anastrozole and tamoxifen was no more effective than either agent alone. There was a statistically significant improvement in disease-free survival at 3 years for anastrozole compared with tamoxifen (hazard ratio (HR) 0.83), but not for combination therapy compared with tamoxifen (HR 1.02). In women with oestrogen-receptor positive tumours, the HR was 0.78 in favour of anastrozole compared with tamoxifen or combination therapy. The recently published Intergroup Exemestane Study did not explore combined treatments, but its results support the sequential use of tamoxifen and AIs. Switching to exemestane after 2–3 years of tamoxifen therapy resulted in a significant reduction in disease-free survival events (HR 0.68; *P*<0.001) compared with continuing on tamoxifen (Coombes *et al*, 2004). Similarly, sequential use of letrozole given after 5 years of tamoxifen showed improved disease-free survival *vs* placebo in the MA17 trial (Goss *et al*, 2003a).

Given that the use of single agents appears preferable, should tamoxifen or AIs be used as first-line treatment in a hormone-therapy naive patient, or in a patient who has completed adjuvant tamoxifen more than 12 months before relapse (i.e. in a potentially tamoxifen-sensitive patient)? The improved efficacy (time to progression) and side effect profile of AIs compared with tamoxifen (Milla-Santos *et al*, 2000; Nabholtz *et al*, 2000; Dirix *et al*, 2001; Mouridsen *et al*, 2001) suggest that these agents should be used first. This is reinforced by the results of studies comparing AIs and tamoxifen in the neoadjuvant setting (Eiermann *et al*, 2001; Ellis *et al*, 2001; Semiglazov *et al*, 2003; Tubiana-Hulin *et al*, 2003). AIs are also considered the agents of first choice for patients with tamoxifen-resistant disease (i.e. those who progress during or within 1 year from adjuvant tamoxifen).

A more problematic choice is faced for a patient who has already received an AI as part of her adjuvant therapy. Is there a role for further treatment with an AI in such a patient? The same question arises at the time of second progression after treatment with AIs in the advanced disease setting.

## RATIONALE FOR SEQUENCING OF AIS

In both cases, the goal of continuing endocrine therapy for as long as possible is to prolong the control of the disease and delay the need for chemotherapy, thus also delaying its associated toxicity. Based on structural differences and different mechanisms of action among the AIs, a lack of cross-resistance between steroidal and nonsteroidal AIs has been hypothesised, thus justifying the use of different AIs in the same patient during the course of her disease.

**Figure 1:**
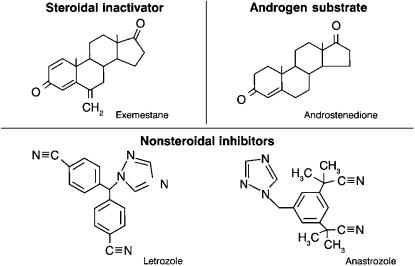
Structural differences between the aromatase inhibitors.

The chemical structures of steroidal (e.g. exemestane and formestane) and nonsteroidal (e.g. anastrozole and letrozole) agents are illustrated in Figure 1. The steroidal inactivator, exemestane, has a very similar structure to the androgen substrate for aromatase, androstenedione (Figure 1); its covalent binding to aromatase results in irreversible inactivation of the enzyme. By contrast, nonsteroidal AIs bind to the haeme part of aromatase, and the inhibition is reversible. It is uncertain, however, whether this structural difference results in significant clinical differences. Both steroidal and nonsteroidal AIs effectively reduce serum oestrogen concentrations in postmenopausal patients. Intratumoural aromatase may, however, be an important additional target for inhibition (Brodie *et al*, 1999), and differences between the AIs at this level cannot be excluded. Moreover, the partial androgen activity of exemestane, which is lacking with the nonsteroidal AIs, may also represent a difference relevant to sequencing. About 70–80% of all breast cancers express the androgen receptor, and androgens have been shown to have antiproliferative effects on breast cancer cell lines (Ando *et al*, 2002).

Although the pharmacological and molecular bases for their lack of cross-resistance need to be investigated further, the possibility of successfully using steroidal and nonsteroidal AIs in sequence is supported by clinical studies (Harper-Wynne and Coombes, 1999; Lønning *et al*, 2000; Carlini *et al*, 2001; Bertelli *et al*, 2002). The largest of these studies, which examined exemestane treatment in patients who had failed on nonsteroidal AIs, found that 6.6% of patients achieved an objective response and 17.4% prolonged disease stabilisation (⩾24 weeks), which translates to a 24% total clinical benefit rate (Lønning *et al*, 2000).

## SELECTION OF THE OPTIMAL SEQUENCE OF AIs

If sequential application of AIs enables prolonged hormonal treatment, in which order should these agents be given? The main choice would appear to be whether to give a steroidal AI before a nonsteroidal AI, or *vice versa*. As yet, clear clinical differences between these two types have not emerged, nor is it known whether their efficacy varies in different patient groups. There may, however, be differences in side effect profiles between the steroidal and nonsteroidal AIs, which may become clearer with further clinical trial results. The main adverse events are hot flushes, gastrointestinal (e.g. nausea and vomiting) and musculoskeletal events. In addition, effects have been reported on bone density, lipid levels and thromboembolic events with hormonal treatments. In the ATAC trial, there was a significantly lower incidence of hot flushes, vaginal bleeding and discharge and venous thromboembolism with anastrozole than tamoxifen, but anastrozole was associated with more musculoskeletal symptoms and fractures (ATAC Trialists Group, 2002). In addition, letrozole has been associated with an increase in bone-resorption markers in plasma and urine (Heshmati *et al*, 2002). By contrast, early results from animal models and postmenopausal volunteers suggest the steroidal AI, exemestane, may have a bone-sparing effect compared with nonsteroidal agents (Goss *et al*, 2001, 2003b). There is also evidence that exemestane does not adversely affect blood lipids (Goss *et al*, 2001; Markopoulos *et al*, 2003), whereas this may not be the case with the nonsteroidal AIs (Elisaf *et al*, 2001). More discussion of effects on bone and lipids is presented elsewhere in the supplement.

The current practice is to use anastrozole or letrozole before exemestane. This is based on the magnitude of the available database and clinical experience with anastrozole and letrozole compared with exemestane. Some evidence, however, suggests that the opposite sequence is equally valid.

### Steroidal AI followed by nonsteroidal AIs

**Figure 2:**
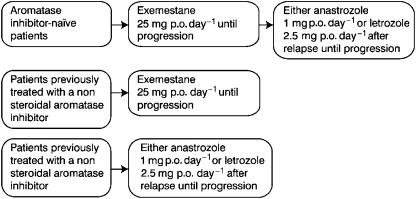
GONO (Gruppo Oncologico Nord Ovest) MIG8 trial: study design.

The GONO (Gruppo Oncologico Nord Ovest) MIG-8 trial was designed to assess treatment with different AI sequences in postmenopausal patients with oestrogen receptor-positive or -unknown advanced breast cancer. This is a prospective, nonrandomised study where patients not previously treated with AIs received exemestane first, and were crossed over to a nonsteroidal AI (anastrozole or letrozole) at the time of progression. Patients with previous exposure to AIs received the alternative AI after entering the study (exemestane for patients pretreated with a nonsteroidal AI; letrozole or anastrozole for patients pretreated with a steroidal AI: Figure 2). Preliminary results have been reported on 10 patients evaluable for the sequence exemestane followed by a nonsteroidal AI, with a clinical benefit rate (objective responses+stable disease ⩾24 weeks) of 40% (Bertelli *et al*, 2002).

Harper-Wynne and Coombes reported on a group of patients (*n*=21) who had received the steroidal AI formestane and were treated with anastrozole (Harper-Wynne and Coombes, 1999). Overall, 62% of the patients stabilised on anastrozole. When only those who had responded to formestane (*n*=12) are considered, 78% achieved further stable disease on anastrozole (Harper-Wynne and Coombes, 1999).

### Nonsteroidal AIs followed by steroidal AI

The other AI sequence assessed in the GONO-MIG-8 trial was exemestane following treatment with a nonsteroidal AI (letrozole or anastrozole). At the first data report, clinical benefit was obtained by 25% among 24 women in this group (Bertelli *et al*, 2002). This is similar to a clinical benefit rate of 20.4% reported by Lønning *et al* (2000) with exemestane in 105 patients after failure of third-generation nonsteroidal AIs. The median duration of clinical benefit was 37 weeks. Interestingly, exemestane was also associated with responses in patients who had not responded to prior hormone therapy (Lønning *et al*, 2000).

Carlini *et al* (2001) conducted a retrospective study on patients receiving formestane after anastrozole or letrozole (*n*=20). The clinical benefit rate was 55%, with a median duration of 15 months.

### Ongoing trials

There are several large trials currently in progress or planned which should throw more light on whether there is an optimal sequence for AI therapy. These include trials of:

- formestane followed by exemestane or anastrozole (SAINT);
- exemestane followed by anastrozole or *vice versa* (GEICAM 2001-03, GONO-MIG-8);
- nonsteroidal AIs followed by fulvestrant or exemestane in patients failing nonsteroidal AIs (SOFEA).

Trials in progress include the NCIC MA27 and the SOFEA trials. NCIC MA27 is a randomised phase III trial comparing exemestane (with or without celecoxib) with anastrozole (with or without celecoxib) for 5 years in preventing cancer recurrence in postmenopausal women following surgery for primary breast cancer. The study aims to recruit 6800 women. The SOFEA trial is comparing fulvestrant with or without anastrozole versus exemestane in 750 patients failing nonsteroidal AIs.

### Other considerations

The impact of new agents, such as fulvestrant, on drug sequencing will also need to be considered. Preliminary results from studies of fulvestrant after progression on tamoxifen and anastrozole reported a clinical benefit rate of 34–50%. Another study has examined fulvestrant in women who had progressed on tamoxifen and an AI (Perey *et al*, 2002). Of 20 patients who had progressed after tamoxifen and a nonsteroidal AI, two showed a partial response and five had stable disease for ⩾24 weeks, which represents a clinical benefit rate of 41%. An open, parallel-group trial compared fulvestrant and anastrozole in postmenopausal women with advanced breast cancer progressing after prior endocrine treatment (Howell *et al*, 2002). Clinical benefit rates were 44.6% for fulvestrant and 45.0% for anastrozole, median time to progression was 5.5 months for fulvestrant and 5.1 months for anastrozole. Similar results were reported in another trial of the same design (Osborne *et al*, 2002).

## CONCLUSIONS

Current evidence suggests that there is clinical utility in using antiaromatase agents in sequence (steroidal inactivators after nonsteroidal inhibitors, or *vice versa*). In other words, antiaromatase drugs of the two types can be used at different times to prolong disease control, before changing to other, less tolerable treatments (such as progestins or chemotherapy). The traditional sequence of hormone therapy, which is to say, adjuvant tamoxifen followed by an AI as first-line therapy at relapse and then progestins as second line could therefore change to adjuvant tamoxifen followed by an AI as first-line therapy at relapse and then another AI as second line and finally, progestins as third-line therapy. The above sequence is likely to change further based on the results of recent and ongoing adjuvant trials (e.g. ATAC, MA-17, BIG 1-98, ARNO, IES, NSABP B33, TEAM), which may result in an increased number of patients exposed to AIs in the adjuvant setting. The choice of first- and subsequent-line hormone therapy for advanced disease will be based on the treatment history of the patient, on the (limited) data reported above on noncross resistance, and on a balance of the expected benefits and toxicities with the agent of choice.

## References

[bib1] Ando S, De Amicis F, Rago V, Carpino A, Maggiolini M, Panno ML, Lanzino M (2002) Breast cancer: from estrogen to androgen receptor. Mol Cell Endocrinol 193: 121–128 12161011 10.1016/s0303-7207(02)00105-3

[bib2] ATAC (Arimidex, Tamoxifen Alone or in Combination) Trialists Group (2002) Anastrozole alone or in combination with tamoxifen versus tamoxifen alone for adjuvant treatment of postmenopausal women with early breast cancer: first results of the ATAC randomized trial. Lancet 359: 2131–2139 12090977 10.1016/s0140-6736(02)09088-8

[bib3] Bertelli G, Garrone O, Merlano M (2002) Sequential use of aromatase inactivators and inhibitors in advanced breast cancer. Proc Am Soc Clin Oncol 21: 60a (Abstract 238)

[bib4] Brodie A, Lu Q, Liu Y, Long B (1999) Aromatase inhibitors and their antitumor effects in model systems. Endocrine-Relat Cancer 6: 205–210 10.1677/erc.0.0060205 10731110

[bib5] Carlini P, Frassoldati A, De Marco S, Casali A, Ruggeri EM, Nardi M, Papaldo P, Fabi A, Paoloni F, Cognetti F (2001) Formestane, a steroidal aromatase inhibitor after failure of non-steroidal aromatase inhibitors (anastrozole and letrozole): is a clinical benefit still achievable? Ann Oncol 12: 1539–1543 11822752 10.1023/a:1013180214359

[bib6] Coombes RC, Hall E, Gibson LJ, Paridaens R, Jassem J, Delozier T, Jones SE, Alvarez I, Bertelli G, Ortmann O, Coates AS, Bajetta E, Dodwell D, Coleman RE, Fallowfield LJ, Mickiewicz E, Andersen J, Lonning PE, Cocconi G, Stewart A, Stuart N, Snowdon CF, Carpentieri M, Massimini G, Bliss JM, Intergroup Exemestane Study (2004) A randomized trial of exemestane after two to three years of tamoxifen therapy in postmenopausal women with primary breast cancer. N Engl J Med 350: 1081–1092 15014181 10.1056/NEJMoa040331

[bib7] Dirix L, Piccart MJ, Lohrisch C, Beex L, Nooij M, Cameron D, Biganzoli L, Cufer T, Yague C, Duchateau L, Lobelle J, Paridaens R (2001) Efficacy of and tolerance to exemestane (E) versus tamoxifen (T) in 1st line hormone therapy (HT) of postmenopausal metastatic breast cancer (MBC) patients (pts): a European Organisation for the Research and Treatment of Cancer (EORTC Breast Group) phase II trial with Pharmacia Upjohn. Prog Proc Am Soc Clin Oncol 20: 29a (Abstract)

[bib8] Eiermann W, Paepke S, Appfelstaedt J, Llombart-Cussac A, Eremin J, Vinholes J, Mauriac L, Ellis M, Lassus M, Chaudri-Ross HA, Dugan M, Borgs M, Letrozole Neo-Adjuvant Breast Cancer Study Group (2001) Preoperative treatment of postmenopausal breast cancer patients with letrozole: a randomized double-blind multicenter study. Ann Oncol 12: 1527–1532 11822750 10.1023/a:1013128213451

[bib9] Elisaf MS, Bairaktari ET, Nicolaides C, Kakaidi B, Tzallas CS, Katsaraki A, Pavlidis NA (2001) Effect of letrozole on the lipid profile in postmenopausal women with breast cancer. Eur J Cancer 37: 1510–1513 11506958 10.1016/s0959-8049(01)00155-1

[bib10] Ellis MJ, Coop A, Singh B, Mauriac L, Llombert-Cussac A, Janicke F, Miller WR, Evans DB, Dugan M, Brady C, Quebe-Fehling E, Borgs M (2001) Letrozole is more effective neoadjuvant endocrine therapy than tamoxifen for ErbB-1 and/or ErbB2-positive, estrogen receptor positive primary breast cancer: evidence from a phase III randomized trial. J Clin Oncol 19: 3808 11559718 10.1200/JCO.2001.19.18.3808

[bib11] Goss P, Grynpas M, Qi S, Hu H (2001) The effects of exemestane on bone and lipids in the ovariectomized rat [abstract]. Breast Cancer Res Treat 69: 224

[bib12] Goss PE, Ingle JN, Martino S, Robert NJ, Muss HB, Piccart MJ, Castiglione M, Tu D, Shepherd LE, Pritchard KI, Livingston RB, Davidson NE, Norton L, Perez EA, Abrams JS, Therasse P, Palmer MJ, Pater JL (2003a) A randomized trial of letrozole in postmenopausal women after five years of tamoxifen therapy for early-stage breast cancer. N Engl J Med 349: 1793–1802 14551341 10.1056/NEJMoa032312

[bib13] Goss PE, Thomsen T, Banke-Bochita J, Hadji P (2003b) Effects of steroidal and nonsteroidal aromatase inhibitors on markers of bone turnover and lipid metabolism in healthy volunteers. 26th Annual San Antonio Breast Cancer Symposium, December 2–6, 2003, San Antonio, Texas (Abstract No. 427)

[bib14] Harper-Wynne C, Coombes RC (1999) Anastrozole shows evidence of activity in postmenopausal patients who responded or stabilised on formestane therapy. Eur J Cancer 35: 744–746 10505035 10.1016/s0959-8049(99)00015-5

[bib15] Heshmati HM, Khosla S, Robins SP, O’Fallon WM, Melton III LJ, Riggs BL (2002) Role of low levels of endogenous estrogen in regulation of bone resorption in late postmenopausal women. J Bone Miner Res 17: 172–178 11771665 10.1359/jbmr.2002.17.1.172

[bib16] Howell A, Robertson JFR, Quaresma Albano J, Aschermannova A, Mauriac L, Kleeberg UR, Vergote I, Erikstein B, Webster A, Morris C (2002) Fulvestrant, formerly ICI 182,780, is as effective as anastrozole in postmenopausal women with advanced breast cancer progressing after prior endocrine treatment. J Clin Oncol 20: 3396–3403 12177099 10.1200/JCO.2002.10.057

[bib17] Lønning PE, Bajetta E, Murray R, Tubiana-Hulin M, Eisenberg PD, Mickiewicz E, Celio L, Pitt P, Mita M, Aaronson NK, Fowst C, Arkhipov A, di Salle E, Polli A, Massimini G (2000) Activity of exemestane (Aromasin®) in metastatic breast cancer after failure of nonsteroidal aromatase inhibitors: a phase II trial. J Clin Oncol 18: 2234–2244 10829043 10.1200/JCO.2000.18.11.2234

[bib18] Markopoulos C, Polychronis A, Fafarelos C, Zobolas V, Bafaloukos D, Papadiamantis J, Misitzis J, Hellenic Breast Surgical Society (2003) The effect of exemestane (Aromasin®) on the lipidemic profile of breast cancer patients: preliminary results of the TEAM trial Greek sub-study. 26th Annual San Antonio Breast Cancer Symposium, December 3–6, 2003, San Antonio, Texas (Abstract 440)

[bib19] Milla-Santos A, Milla L, Rallo L, Solana V (2000) Anastrozole *vs* tamoxifen in hormonodependent advanced breast cancer: a phase II randomized trial. Breast Cancer Res Treat 64, (Abstract 173) 10.1023/a:1006440802709 11261827

[bib20] Mouridsen H, Gershanovich M, Sun Y, Perez-Carrion R, Boni C, Monnier A, Apffelstaedt J, Smith R, Sleeboom HP, Janicke F, Pluzanska A, Dank M, Becquart D, Bapsy PP, Salminen E, Snyder R, Lassus M, Verbeek JA, Staffler B, Chaudri-Ross HA, Dugan M (2001) Superior efficacy of letrozole versus tamoxifen as first-line therapy for postmenopausal women with advanced breast cancer: results of a phase III study of the International Letrozole Breast Cancer Group. J Clin Oncol 19: 2596–2606 11352951 10.1200/JCO.2001.19.10.2596

[bib21] Nabholtz JM, Buzdar A, Pollak M, Harwin W, Burton G, Mangalik A, Steinberg M, Webster A, von Euler M (2000) Anastrozole is superior to tamoxifen as first-line therapy for advanced breast carcinoma in postmenopausal women: results of a North American multicenter randomized trial. J Clin Oncol 18: 3758–3767 11078488 10.1200/JCO.2000.18.22.3758

[bib22] Osborne CK, Pippen J, Jones SE, Parker LM, Ellis M, Come S, Gertler SZ, May JT, Burton G, Dimery I, Webster A, Morris C, Elledge R, Buzdar A (2002) Double-blind, randomized trial comparing the efficacy and tolerability of fulvestrant versus anastrozole in postmenopausal women with advanced breast cancer progressing on prior endocrine therapy: results of a North American trial. J Clin Oncol 20: 3386–3395 12177098 10.1200/JCO.2002.10.058

[bib23] Perey L, Thürlimann B, Hawle H, Bonnefoi H, Aebi S, Pagani O, Goldhirsch A, Dietrich D (2002) Fulvestrant (‘Faslodex’) as hormonal treatment in postmenopausal patients with advanced breast cancer progressing after treatment with tamoxifen and aromatase inhibitors. Breast Cancer Res Treat 76(Suppl 1): S72 (Abstract 249)

[bib24] Semiglazov VF, Semiglazov VV, Ivanov VG, Zitzova EK, Dashyan GA, Kletzel A, Bozhok AA, Nurgaziev KS, Tzyrlina EV, Berstein LM, Petrov NN (2003) Neoadjuvant endocrine therapy: exemestane (E) *vs* tamoxifen (T) in postmenopausal ER+ breast cancer patients (T1-4N1-2MO). Breast Cancer Res Treat 83(suppl 1): S22 (Abstract 111)

[bib25] Tubiana-Hulin M, Spyratos F, Becette V, Mauriac L, Romieu G, Bibeau F, Bieche I, Bourgeois H (2003) Phase II study of neo-adjuvant exemestane in postmenopausal patients with operable breast cancer. Breast Cancer Res Treat 83(suppl 1): S106 (Abstract 443)

